# Are Mid to Late Adolescents with Asthma Ready for Transition of Care? A Qualitative Study

**DOI:** 10.3390/children9101573

**Published:** 2022-10-18

**Authors:** Hyekyun Rhee, Lindsay Batek, Tanya Wallace-Farquharson, Laurene Tumiel-Berhalter

**Affiliations:** 1 School of Nursing, University of Texas at Austin, 1710 Red River St., Austin, TX 78712, USA; 2 School of Nursing, University of Rochester, 601 Elmwood Ave., Box SON, Rochester, NY 14642, USA; 3 Jacobs School of Medicine and Biomedical Sciences, University of Buffalo, 77 Goodell St., Buffalo, NY 14203, USA

**Keywords:** adolescents, asthma, transition readiness, self-management, parent roles, provider communication

## Abstract

This qualitative descriptive study explores experiences and perspectives of mid-to-late adolescents about growing up with asthma, and the roles of parents and providers as they transition. Purposeful sampling was used to recruit and enroll adolescents aged 16–20 years with asthma. Forty-one adolescents participated in a focus group or individual interview, and content analysis was conducted to analyze the data. The mean age of participants was 17.7 years, the majority (56%) of whom were Black. Themes that emerged included concerns about becoming an adult with asthma and its self-management, parental involvement, and communication with providers. Adolescents felt burdened by asthma, few considered becoming adults with asthma, and their future outlook was pessimistic with concerns related to worsening symptoms, inadequacy in symptom self-management and limitations on career choices due to asthma. Deficiencies in self-management were noted, parents still played major roles in adolescents’ asthma care, and transition of care was seldom discussed with the providers. Mid-to-late adolescents with asthma are inadequately prepared for transition of care, and parents and providers insufficiently engage adolescents in the preparation. Parent, provider, and adolescent partnership is critical to achieve adolescent readiness for independence in asthma management and to ensure proper asthma care continuity post transition.

## 1. Introduction

Asthma represents a common health concern in many adolescents, with 8.7% (>4 million) of US adolescents reporting a current asthma diagnosis in 2019 [1]. The disease continues to be an ubiquitous health threat in young adults with nearly 10% of people aged 20–24 years having reported current asthma in 2019 [1]. Nearly 70% of adults with severe asthma reported having had asthma symptoms before the age of 20 [2]. As such, under- or untreated pediatric asthma can potentially progress to active or serious disease as an adult [3]. Therefore, preventing disease progression through uninterrupted adequate treatment beyond adolescence is critical to ensure healthy and productive life for those with asthma.

Young adults with asthma often experience discontinuity in their asthma care (e.g., fewer or no follow-ups, long lapse in prescription medications) as they transition from adolescence to adulthood [4,5,6,7]. Although the extent to which such discontinuity directly contributes to asthma-related morbidity or quality of life in young adults remains largely unknown, increased high-acuity emergency visits and associated costs among young adults with a chronic condition including asthma have been documented after transfer from pediatric to adult care [8]. Similarly, literature reports declining health in young adults with chronic conditions in association with transition from pediatric to adult health services [9,10,11,12,13].

Despite asthma being the most prevalent chronic condition in adolescence and young adults, and the serious health implications of inadequate transitional asthma care, overall perceptions of mid to late adolescents (MLAs) with asthma transitioning to independent young adults are largely unknown. MLAs aged 16–20 years represent adolescents who are legally allowed to drive in the US, capable of carrying out adult responsibilities, and at the verge of the transfer of care or in the process of transitioning. To date, two qualitative studies have been conducted to explore the self-management goals of U.S. adolescents (14–18 years-old) with asthma as they transition to adult healthcare [14] and to describe the general feelings felt by Swedish young adults (24 years-old) with asthma retrospectively during their transition period [5]. However, no studies specifically target mid-to-late adolescents’ unique perspectives on becoming adults with asthma, parents’ roles in asthma care during the transition and their communication with the providers about the transition. The purpose of this qualitative study is to explore the MLAs’ perceptions and experiences about (1) growing up with asthma, (2) parents’ involvement in asthma care and (3) communication with the providers about transition of care.

## 2. Materials and Methods

### 2.1. Study Design and Study Sample

A qualitative descriptive study was conducted in 2019 to explore the experiences and perceptions of mid-to-late adolescents with asthma about transitioning to adult-focused healthcare. A purposeful sampling method was used to recruit and enroll adolescents (16 to 20 years of age) with a current diagnosis of asthma for at least 1 year, for which a controller medication was prescribed. Adolescents with a comorbid condition requiring daily medication were excluded as it can alter transition experiences. One half of the sample were the participants of the first author’s previous study, and the other half were recruited from local colleges, community centers, clinical practices, and word of mouth. Detailed sampling methods are provided elsewhere [15].

### 2.2. Study Procedure and Data Collection

The study protocol was approved by the Institutional Review Boards (IRBs) in the academic institutions where the study was originated and where data were collected. Written informed consent was obtained from participants >18 years, while parent permission and adolescent assent were obtained for those ages 16–17 years old. Focus groups were initially offered. However, due to practical challenges in coordinating adolescents’ availability for the focus groups, the original protocol was amended and approved by the IRBs to offer an option of individual interviews. Focus groups were conducted for 16–17-year-olds and 18–20-year-olds separately to distinguish minors from non-minor participants. A total of 5 focus groups were conducted including 3 for younger and 2 for older participants, and each group was attended by 2–4 participants. Twenty-four individual interviews were conducted, 12 each for younger and older participants. For both focus groups and individual interviews, we used semi-structured interview questions (Table 1).

Because all interviews were audio recorded, we used participants’ pseudonyms to protect privacy. Audio recordings were transcribed verbatim, which was subsequently verified for accuracy by a research staff person not involved in transcription.

### 2.3. Data Analysis

Descriptive statistics were used to analyze demographic data. Interview transcriptions were coded and analyzed in Microsoft Word using qualitative content analysis techniques [16,17]. Initially, responses to each question were labeled with short phrases using the quotation mechanism. Then, codes were developed by consolidating quotations into main categories of responses. A simple coding guide with code definitions were developed, and then used to code the data using a color-coding method. Two members of the research team initially analyzed the data independently and compared the coding and supporting quotations. Any discrepancies in the analysis were reconciled through discussion. A third member conducted separate analyses by using the coding guide initially developed. Again, any discrepancies with the first round of analyses were discussed and resolved upon mutual agreement. Triangulation by involving multiple investigators in data interpretation was supported as a way to establish the validity and reliability of qualitative data analysis [18]. Whenever applicable, we counted the number of respondents to each response category to show majority and minority opinions in the responses. Data from focus groups and individual interviews were analyzed separately and compared for similarities and dissimilarities in codes. Because no differences in codes were found, the following report describes the consolidated results.

## 3. Results

### 3.1. Sample Characteristics

This study included 41 participants. The mean age of participants was 17.7 years, and the majority (56%) represented a racial minority, predominantly black (83%). Twenty adolescents had a public health insurance, and 22 reported to have had asthma diagnosed at the age of 6 or earlier. Table 2 summarizes the sample characteristics.

### 3.2. Living with Asthma

The majority reported ongoing difficulties living with asthma, which provoked many emotional reactions such as “hard”, “upsetting”, “annoying”, “scary”, “panicking”, and “frustrating”. These negative emotions were triggered by several challenges, most frequently related to limited daily or sports activities, as stated, “…it [asthma] limits me, doing exercise, working out”. The second most common challenge mentioned was associated with ongoing responsibilities of asthma management.

[I]t was very hard, probably, for me to manage…because it was a lot of responsibility… just to always have to leave the house and remember to grab my inhaler, all that.

(female, 17 year-old, White)

Although for some asthma became less acute as they grow older, many still reported high levels of symptoms in association with the season or weather, or comorbid conditions including obesity, allergies, or bronchitis. In those teens, the unpredictable and uncontrollable nature of symptoms was recognized as a major challenge.

It’s kind of scary because you never know what’s like gonna happen. Because I could easily have an asthma attack or you know have to be hospitalized because my asthma is acting up.

(female, 19 year-old, Black)

Some participants expressed a sense of being different as a reason for a hardship as captured in the following:

I kind of grow different from kids who didn’t have asthma. You know it’s like, having it just puts you in a different place than someone who doesn’t have it.

(female, 19 year-old, Black)

Somewhat neutral or optimistic attitudes toward asthma were expressed by a few who “learned to deal with it [asthma]”, or “got used to it [asthma]”. Those who “learned to deal with it” tended to perceive asthma as a controllable or manageable condition despite ongoing, at times intense, symptoms. Those who “got used to it” accepted asthma as an unavoidable part of their lives stating that “it’s constantly there” or “I just live the same”. Ironically, some who perceived asthma no longer as a threat reported not taking asthma medication, despite occasional severe symptoms.

### 3.3. Becoming Adults with Asthma

Half of the participants responded that they had not thought about becoming adults while still having asthma (“It hasn’t crossed my mind”) while the other half had thought about it with a rather pessimistic outlook (“I accepted it”) of asthma staying the same. Most participants expressed concerns about progressively deteriorating symptoms and their inability to manage symptoms as a grown-up.

[I]t seems that my asthma gets progressively worse.

(female, 19 year-old, White)

Living alone and if I can’t reach my asthma pump in time. That’s what worries me.

(male, 17 year-old, unknown race)

Many lamented about asthma limiting their daily lives and future career plans.

[S]ome of the stuff that I wanna do, I have to be very careful doing it, because certain stuff is like hard for asthma attacks.

(female, 17 year-old, Black)

If you want to be like an active worker like a cop, a firefighter, a personal trainer or a runner or like an athlete you will know that you can’t really do that because you have asthma.

(male, 16 year-old, Black)

Other concerns included passing on asthma to their offspring (“my future kids will have it”) or causing inconvenience to others (“I don’t wanna keep them up [with my coughing]”).

Only few participants denied having any concerns about asthma affecting their future, anticipating that they would “grow out of it [asthma] eventually” or asthma would “become less severe or better controlled”.

### 3.4. Asthma Self-Management

A few participants reported taking controller medication, pre-exercise treatment, and maintaining good health as ways to prevent symptoms. Monitoring symptoms and triggers were rarely brought up. Many were unaware of their triggers or symptoms, only 7 participants accounted for their triggers or symptoms. However, knowing triggers or symptoms does not always translate into actions to manage them as stated as the following:

I mostly know most of the things that trigger my asthma, and I just kind of…most of the time I wait to see how long my symptoms persist for and I kind of act accordingly.

(female, 16 year-old, Black)

For symptom management, the majority mentioned “carrying an inhaler”, and only 4 specifically reported using a rescue inhaler when symptoms occur, while taking deep breaths or resting were the only remedy taken for some. Only 5 older and 2 younger participants responded that they engaged in actions to (re)fill prescriptions, including “keep track [of medication refill]”, “call in medication”, and “get my med on my own”.

Most responded that they gradually became more independent in the last 2–3 years in managing asthma, while a few perceived no changes. Many did not take medication as much as they used to for various reasons, including “get[ting] lazy”, “don’t feel as important”, “don’t need to take it”, “asthma don’t affect me no more”, or simply “I don’t want to”. Participants who did not feel the need of medication would rather control symptoms by limiting their activities or non-pharmacological methods.

I know how to control like…not getting to the point where need to grab my inhaler. I know my restrictions.

(female, 20 year-old, Black)

I don’t use it [inhaler] as much now, because I know how to control it. When I’m running or when I’m wheezing, I’ll stop and take deep breaths and I’ll sit down for a couple seconds until I feel better.

(female, 17 year-old, Black)

Overall, participants expressed confidence in managing asthma independently. For many, confidence was not a dichotomous attribute either presence or absence. Rather, it was commonly understood as a spectrum, within which the levels of confidence could fluctuate (“ups and downs”) or undergo a set-back (“I just fall off”) as captured in the following statement:

I’m confident but I’m not fully confident because I have a tendency to go off… Because I’ll say one minute ‘okay I’ll take my medicine at this time’ and then I’ll completely forget or I’ll-I’ll just push it off to the side. So I’ll say a 7 or 8 [out of 10] because I’m not quite yet confident but I’m also not lacking too much confidence.

(female, 16 year-old, Black)

Particularly limited confidence was expressed in taking medication, making clinical appointments, filling prescriptions, and making others (e.g., friends, teachers, and coaches) understood about their asthma. Participants felt confident when they “know a lot” about medication, symptoms and making appointments and when “perform the [management] actions” independently. Confidence was also felt when they were “organized” and “reminded” to take medication. For some, confidence was based on the dependability of parent’s involvement.

I feel confident…because like the way it is like whenever I don’t take it [medicine] like before I leave out the door, my mom will be like you should take your medicine and that’s when I’ll take it.

(male, 16 year-old, Black)

### 3.5. Parental Involvement in Asthma Management

The most frequently mentioned current parental role in managing asthma was “reminding” to take medication and monitoring conditions as stated, “she [my mom] comes to check on me…see if I’m alright”. Parents were also said to play the roles of teaching and coaching teens in managing asthma. For a few participants, parents would take on more active roles in managing symptoms by providing medication and comfort and controlling indoor triggers. For the majority, parents played major roles in “making sure it’s [medication] filled on time before I run out”, and “taking me to the doctor”. Parents’ over-involvement in asthma management was also noted by two younger participants, stating “my mom is really overprotective about it [asthma]…Now, my mom does everything”. Only three participants (2 older and 1 younger) denied any parental involvement in their asthma care.

Many participants reported no conflicts with the parents related to their asthma management, although parents’ distrust of their teens sometime resulted in an argument. Some older participants expressed their frustration over parents’ involvement stating, “[I] don’t really like when people [parents] tell me what to do”, while others were annoyed by their parents’ repeating reminders, saying “she’d be like ‘I told you to take your meds’… yeah you did, like 5 times”. Such negative reactions would lead them to disregard parents’ advice (e.g., “she just want[s] me to start taking it serious, I don’t take it serious”). Some participants wished that their parents be “more hands-off” or patient, as elaborated in the following:

I would kind of prefer that she waited until I said I was running low because I tell her in advance about things of me… But with asthma she just comes to me, instead of letting me come to her about it.

(female, 16 year-old, Black)

Nonetheless, most participants wanted their parents to continue to be involved in their asthma management, admitting that they “still need somebody to rely on a little bit”.

I still want her [mom] to like, like if I do move out, I still want her to come, like, check on me or call me… and just to see if I’m awake or make sure I’m alive

(male, 17 year-old, Black)

[I]f it [inhaler] wasn’t just there I probably wouldn’t use it because I wouldn’t have it…if I didn’t have my inhaler which I wouldn’t have had it…if it wasn’t for her [mom], I would been in much worse situation

(male, 18 year-old, White)

Many participants did not envision notable changes in the level of their parents’ involvement in the next 2–3 years. Younger participants expected that parents would continue to play active roles simply out of fear, while some older participants felt that they would continue to be independent with no to minimal parental involvement in asthma care.

I think she’s [mom] scared to let me go by myself cause she know I’m not gonna take my medicine.

(female, 17 year-old, Black)

Some participants, both younger or older, anticipated parental involvement to diminish or stop in 2–3 years primarily because of their “moving out” of parental residence, their taking “more responsibility” of asthma care and/or their asthma becoming “better controlled”.

### 3.6. Communication with Providers

Although most claimed to have communicated directly with their providers, 11 participants, including two older teens, admitted that their parents mostly communicated with the providers on their behalf. Parents’ presence during an appointment negatively affected their level of comfort in communicating with the providers.

I would feel more comfortable when the parent not in the room… there’s some things you’d rather share just with your doctor, not with your parent.

(male, 19 year-old, White)

Some participants observed that parents often shared with the providers inaccurate information concerning their asthma, while others stated that the providers tended to speak with the parents rather than the teens.

[P]arents like to put their opinion into everything. And a lot of their opinions don’t even be right”.

(female, 17 year-old, Black)

[T]hey’ll [providers] talk to my parents about me while I’m sitting right there.

(female, 20, Biracial)

However, parents dominating the conversation with the providers was not always perceived negatively as some viewed it necessary because they often forgot to share important information or when the conversations were about medications or triggers. Nonetheless, the presence of parents during the appointment was discussed extensively as a “barrier” to their communication with the providers.

A range of communication patterns emerged from teen speaking “not a lot” to actively engaging in conversation with their providers by asking questions or sharing information. Frequent topics of conversation were about symptoms and medications. Providers engaged adolescents in conversation primarily by asking questions and providing guidance mainly about medication use.

For the majority, the topics of their becoming independent or transitioning to adult practice had never been brought-up during appointments. Of eight participants who had discussed the transition with providers, five were older adolescents (18–20 years), of which three had already completed their transition to adult care. Only two older participants had had a conversation with their provider about health insurance. Most participants felt comfortable, safe, or at ease communicating with their providers with whom they were familiar with and felt respected and cared for. In contrast, some teens perceived not fully being understood by their providers, hindering their communication.

I felt like what I said wasn’t well received. Or they didn’t fully digest what I said. And that was my first one-on-one. And that’s why it kind of bothered me.

(male, 19 year-old, White)

Despite the overall denial of any concerns related to the transition of care, many participants expressed their apprehension about changing doctors or treatment plans, worsening symptoms, limited knowledge about asthma management, and their inability to manage triggers or carry out asthma-related responsibilities independently.

## 4. Discussion

This study explored the experience and perceptions about growing up with asthma and transitioning to adult healthcare in mid-to-late adolescents (MLAs). MLAs continue to be burdened by symptoms limiting activities, disease management responsibilities and unpredictable nature of symptoms. In fact, activity limitation is a common asthma-related impairment with over 50% of teens with asthma affected by it [19,20,21]. These challenges posed by asthma appear to be the continuous source of hardship and negative emotions among MLAs, such as annoyance, fear, panic, frustration and feeling of being different. The negative emotional responses to asthma align with previous studies reporting the increased risk of psychological difficulties in young people with asthma [22,23,24,25,26].

It is also noteworthy that thoughts of becoming adults with asthma had never occurred to most participants. Similarly, an earlier study reported that only 35% of youth aged 16–25 years had thought about transitioning to adult healthcare [27]. A few who had ever thought about becoming grown-ups with asthma offered mostly a bleak prospect with expressed concerns about deteriorating symptoms and their inability to manage asthma successfully as well as asthma limiting their career choices or passing asthma onto their children. Either complete disregard of or heightened concerns about becoming adults in adolescents with asthma suggests inadequate transition readiness among MLAs, underscoring the need for an intervention.

We found that MLAs’ knowledge on asthma self-management was rudimentary or superficial at best with carrying and/or taking an unspecified inhaler often mentioned as an only measure to manage asthma by most participants. Similarly, adolescents’ limited knowledge on asthma self-management and medication has been documented [28,29,30]. As in other studies of adolescents [30,31,32], some participants’ resorting to solely relaxation techniques to relieve symptoms raises a concern. It is also noteworthy that monitoring or managing triggers was seldom recognized as part of asthma self-management, which is in line with earlier publications [29,33,34] reporting adolescents’ general lack knowledge about the triggers and their proper management. Because environmental triggers are closely linked to high asthma morbidity [35,36,37], adolescents’ failure to recognize trigger management as important part of asthma self-management is concerning. Despite our sample at the juncture of transitioning to adulthood and adult-focused care period, (re)filling prescription independently was not performed by the majority. This finding is consistent with other studies demonstrating general lack of readiness in prescription management in adolescents and young adults [27,38,39]. Given one’s capacity to self-manage disease being critical to gauging and assuring transition readiness in adolescents and young adults [9,40,41], the notable deficiencies in self-management in our participants are worrisome and warrant an intervention to improve self-management knowledge and skills.

Nearly all participants responded that they had become independent in managing asthma as they got older. Ironically, the independence often led them to become less adherence to treatment. Likewise, study have documented inverse relationships between medication adherence and age in adolescents [42,43,44]. Our mid-to-late adolescents chose not to take medication for a variety of reasons including misperceptions about medication, overestimation of asthma control, personal preference or simply being complacent. It is also noteworthy that some would restrict their activities in order not to feel the need of medication. In such cases, activity limitation should be viewed as a proactive measure used by adolescents to control symptoms rather than an indication of uncontrolled asthma. Notwithstanding, most participants were highly confident in managing asthma independently despite their stated poor treatment adherence. Similarly, adolescents’ tendency to overestimate their capacity to independently manage a disease has been reported [40], and their high confidence was not linked to greater degree of self-care or preparedness for transition [27]. Interestingly, our MLAs viewed confidence in a spectrum that varies depending on the tasks of asthma management or circumstances. Limited confidence was noted specifically in managing clinical appointments and prescriptions and communicating with others about their asthma, which are critical skills for adolescents to successfully transition to adult-focused care. Thus, a proper transition intervention to boost the confidence and skills in these areas of self-management is needed.

Consistent with previous studies of adolescents [45,46], parents were invariably reported as critical players in most MLAs’ asthma management with some variations in the degree and types of involvement. As in other publications [14,27,46,47], common parental roles involve reminding to take medication, teaching/coaching, filling prescriptions and accompanying to the appointments. In most cases, parental involvement itself did not cause conflicts with their teens, but parents’ distrust did. Surprisingly, most including the older participants admitted that they would want and need parents’ continuous involvement in their asthma management, although the degree might diminish with time. Coupled with parents’ general hesitancy to relinquish responsibilities of disease management to their teens [48], the absence of strong motivation in adolescents to be independent of their parents in managing asthma could potentially hamper timely and successful transition into adult care. Only a few participants became turned off by being told by their parents and their repeated reminders. These adolescents tended to disregard parents’ advice as reported in other studies [25,46,49], and wished for parents’ patience and “hands-off” approach. The findings highlight the importance of early transition planning that involves training parents to gradually and strategically negotiate and delegate the responsibilities of asthma management to adolescents early on, along with the provision of necessary supervision and support. This will increase the likelihood of adolescents achieving greater competency and independence in managing asthma in time for transition.

Although most participants responded having communicated with their providers directly to varying degrees, some including older participants still relied on their parents in communicating with the providers. Adolescents seemed ambivalent about parents’ presence during the appointment, which was perceived necessary to ensure sharing critical information with the providers and simultaneously as a major barrier to adolescents’ direct or effective communication with the providers. Given poor parent-adolescent agreement in asthma symptom reports with adolescents reporting more symptoms [50,51], however, relying on symptom information communicated by parents for their teens could potentially result in undertreatment of the disease.

Providers appear to engage adolescents in conversation around medication and symptoms, yet topics on independent management of asthma or the transition of care were rarely brought up as reported in an earlier study [27]. This suggests that most adolescents receive inadequate guidance or support from their providers related to care transition, despite various concerns from switching providers to taking full responsibilities of asthma management as reported by our participants and in the literature [52]. Such neglect of the providers to identify and address the needs related to impeding transition in conversation with adolescents may have contributed to widespread deficiencies in transition readiness in young people with chronic conditions [53,54]. Adolescents’ positive relationships and better communication with their providers have been found to be associated with better adherence to self-management [33,55,56,57,58,59], and greater motivation to take medication independently in adolescents with asthma [60,61]. Therefore, providers’ efforts to establish and maintain positive and trusting relationships with adolescents through good communication skills (e.g., listening and empathizing) is important to enhancing adolescents’ self-management that is essential to successful transition.

Caution is warranted in interpreting findings given several limitations of the study. First, because study participants are limited to mid-to-late adolescents with asthma, applying the findings to younger adolescents or those with other chronic conditions requires extreme care. Second, this study was not designed to compare transition experiences in different race groups. Therefore, no notable racial differences in transition experiences in this study should not be construed as no differences in transition needs between White and adolescents from other race groups. Third, because participants’ responses were not linked to their asthma control, it is unknown the degree to which their perceptions or experiences were affected by differing symptom severity. Lastly, information on parents’ roles and adolescent-providers communication was based entirely on adolescents’ perspectives. Thus, it may not be the accurate reflection of reality.

## 5. Conclusions

Mid-to-late adolescents with asthma appear to be in great need as they transition into young adults and adult-focused care. Lessons gleaned from the study along with recommended actions for clinicians are summarized in Table 3.

Asthma continues to pose serious threats to the well-being of mid-to-late adolescents who simultaneously deals with various concerns in relation to moving into adulthood. A noted paradox between actual deficiencies and perceived high confidence in asthma self-management in this age group is particularly concerning and needs to be addressed. Despite the developmental expectation of achieving independence from parents, many still depend on their parents in a wide range of tasks of asthma management from medication taking to communicating with their providers, albeit variations in degree depending on the age. Indeed, parents are still relevant and important players in asthma self-management of mid-to-late adolescents, which necessitates the inclusion of parents in any care transition planning for adolescents with asthma. Adolescents’ denial of having discussed the matters of impending care transition with their providers is alarming. Because of time constraints, addressing the extensive topics and needs associated with the transition during clinical appointments may be impractical. Therefore, offering access to other resources within the healthcare system (e.g., patient educators and case managers) seems necessary to facilitate the transition process while preparing adolescents and their parents for the transition. Alternatively or additionally, technological solutions catering to the specific needs of transitioning adolescents with asthma could be a developmentally appealing, sustainable and potentially cost saving approach to assist successful transition of care.

## Figures and Tables

**Table 1:** Semi-structured interview questions.

	Questions
Overall experience and perspectives	▪ What has it been like for you to live with asthma?
	▪ Have you thought about becoming an adult while still having asthma? What worries/excites you most when you think about becoming a grown-up with asthma?
	▪ Has anyone in your doctor’s office discussed with you and your parent about being independent in asthma care and switching to adult practice? If yes, what specifically did your doctor tell you and your parent about transitioning to adult care?
	▪ What are your concerns in relation to the transition?
Transition readiness and needs	▪ What role do you play currently in your asthma management? How has that changed over the last 2–3 years?
	▪ How much do you communicate directly with your provider (instead of your parent) about your asthma during a clinic appointment? What information do you share with your provider? - If you do communicate directly with your provider, how comfortable are you with it? Would you like it to be different? How? - How do you feel about the provider interacting with you? What do they do to involve you in your care?
	▪ How confident are you that you could carry out the tasks of asthma management (e.g., remembering to take asthma meds, monitoring and taking care of asthma triggers, making a clinical appointment, filling the prescription, etc.) on your own when you are no longer a teenager?

**Table 2:** Sample characteristics.

	N	%
Age (years)		
16–17	20	48.8
18–20	21	51.2
Gender		
Male	17	41.5
Female	24	58.5
Race		
White	16	39.0
Black, bi/multi-racial, Hispanic	23	56.1
Missing	2	4.9
Health insurance		
Private	16	39.0
Public	20	48.8
Missing	5	12.2

**Table 3:** Lessons learned and recommendations for clinical practice.

Findings/Lessons	Recommended Course of Actions
▪ Adolescents experience negative emotional reactions to asthma and treatment responsibilities are common.	▪ Identify and assess negative emotions; determine the degree to which such negative reactions are associated with asthma control and treatment adherence.
▪ Adolescents have various concerns about becoming adults with asthma.	▪ Explore the nature of concerns, and target to address the specific concerns during the transition period.
▪ Adolescents know little about asthma triggers, symptoms and medication.	▪ Identify knowledge gaps in asthma triggers, symptoms and treatment, and address the gaps through targeted education.
▪ Adolescents feel unprepared to manage prescriptions.	▪ Establish step-by-step plans in collaboration with parents to prepare adolescents to manage their own prescriptions.
▪ Adolescents do not adhere to treatment for a variety of reasons.	▪ Explore and address underlying reasons for poor treatment adherence.
▪ To adolescents, confidence in asthma management is not a constant attribute.	▪ Conduct periodic assessment of self-efficacy in asthma self-management.
▪ Parents are still a major player in adolescents’ asthma management.	▪ Assess the type and degree of parental involvement in adolescents’ asthma care. Plan and assist parents’ strategic delegation of asthma care responsibilities to adolescents from early on.
▪ Adolescents perceive negatively about their parents’ presence or dominating conversation during a clinical appointment.	▪ Set aside a time to meet with adolescents alone during an appointment.
▪ Adolescents still depend on parents for sharing asthma information with providers.	▪ Educate and train adolescents to be an effective communicator and advocator for their health.
▪ Providers rarely engage adolescents in conversations about becoming independent and transitioning into adult-focused healthcare.	▪ Assess periodically the degree of independence in managing asthma and transition readiness. Provide necessary education and resources to facilitate successful transition of care.
▪ Adolescents’ perception of not being understood by the providers hinders teen-provider communication.	▪ Establish and maintain positive, trusting relationships with adolescents by exercising good communication skills (e.g., listening and empathizing).

## Data Availability

The data presented in this study are available on request from the corresponding author. The data are not publicly available due to privacy concerns.
